# Increased organic fertilizer significantly increases leaf nitrogen and phosphorus but not carbon content in a tropical tea plantation

**DOI:** 10.1038/s41598-025-11057-z

**Published:** 2025-07-19

**Authors:** Jing-li Lu, Ying Wang, Di Li, Qiu Yang, Yamin Jiang, Peng Wang, Tianyan Su, Geming Li, Qian Shi, Huai Yang, Wenjie Liu, Mengyang Fang

**Affiliations:** 1 Center for Eco-Environment Restoration Engineering of Hainan Province, School of Ecology, Hainan University, Haikou, 570228 People’s Republic of China; 2 Institute of Tropical Bamboo, Rattan and Flower, Sanya Research Base, International Center for Bamboo and Rattan, Sanya, 572000 People’s Republic of China; 3 Sanya Tropical Ecosystem Carbon Source and Sink Field Scientific Observation and Research Station, Sanya, 572022 People’s Republic of China

**Keywords:** Tropical tea plantation, Carbon, Nitrogen, Phosphorus, Stoichiometry, Agroecology, Biogeochemistry

## Abstract

Carbon (C), nitrogen (N), and phosphorus (P) are essential elements for plant growth and metabolism, with their stoichiometry playing a pivotal role in ecosystem functioning. In tea plantations, fertilization strongly influences the balance of these elements. However, the effects of organic fertilizers on the leaf C, N, and P contents and their stoichiometry in tropical tea plants remain poorly understood. In this study, we examined the impact of organic fertilizer on the leaf C, N, and P contents and their ratios of *Camellia sinensis* var. *assamica* (CSA) and *Camellia sinensis* var. *sinensis* (CSS) in Hainan, China. Our findings indicated that organic fertilizer significantly increased leaf N and P contents but had no effect on leaf C content. Additionally, organic fertilizer significantly decreased leaf C: N ratio of CSA and CSS, as well as leaf C: P ratio of CSA, but significantly increased leaf N: P ratio of CSS. Soil organic carbon (SOC), total N, and total P also increased, while their stoichiometric ratios decreased in CSA but not CSS. In the unfertilized tea plantation, leaf C, N, and P contents and their stoichiometry of CSA and CSS were significantly correlated with soil NO_3_^−^-N, whereas they were significantly correlated with soil NH_4_^+^-N in the fertilized tea plantation. Our results suggest that the differential responses of leaf C, N, and P contents of CSA and CSS to organic fertilizer might be attributed to the distinct leaf physiological characteristics of these two tea plant species.

**Supplementary Information:**

The online version contains supplementary material available at 10.1038/s41598-025-11057-z.

## Introduction

Carbon (C), nitrogen (N), and phosphorus (P) are the most essential elements for plants development and influence their metabolic processes^1^. Specifically, carbohydrates provide the basis for plant growth, reproduction, and structure, which constitute approximately 50% of the dry weight of plants^2^. In addition, these carbohy1drates not only support plant growth but also provide C skeletons for the N assimilation process^3^. N is a fundamental component of enzymes and chlorophyll in plants, and plays a key role in controlling C assimilation and primary productivity^4^. P is a key element in ribosome production and an essential component of RNA, DNA, and ATP^5^. The availability of P directly affects ATP production, which in turn influences the fixation and metabolism of C and N^6^. Therefore, plant growth and reproduction depend not only on the availability of individual nutrients but also on the balance between various elements^7^. That is, nutrient elements are mutually coupled in the biochemical functions of plants^8^.

Ecological stoichiometry refers to the ratios of important chemical elements (e.g., C: N:P) in ecological interactions and processes^9^, and it is an effective tool for exploring the balance and cycling of coupled elements^10–12^. Plant C: N and C: P ratios reflect their ability to absorb and accumulate C and the degree of nutrient utilization, which can indicate plant growth rate^13^. The plant N: P ratio has been widely used to assess nutrient limitation, with N: *P* < 14 indicating nitrogen limitation and N: *P* > 16 suggesting phosphorus limitation^14–16^. Leaves are the primary photosynthetic organs of plants and the main interface for material exchange between plants and the ambient environment, making them highly sensitive to environmental changes^17^. Leaf C: N:P stoichiometry can directly reflect the efficiency of resource utilization by plants, aiding in the understanding of plant adaptation strategies under different environmental conditions. Most studies have focused on leaf C: N:P stoichiometry in natural ecosystems^18–21^, while relatively few studies have been conducted in agricultural ecosystems.

Tea plantation is a specific type of agricultural ecosystem, whose establishment and development are entirely dependent on anthropogenic activities. China is the world’s largest tea-producing country, with the tea plantation area of 3.22 million hectares, accounting for approximately 62.1% of the global tea plantation area^22^. Tropical tea plantations account for approximately 6–7% of total tea plantation area in China^23^. Although their proportion is relatively limited, tropical tea plantations hold irreplaceable economic value in the development of the tea industry and play a significant ecological role within terrestrial ecosystems. For tea plantations maintained by artificial management, excessive anthropogenic disturbances, such as fertilization, pruning, and picking, result in significant differences in the physiological characteristics and resource use efficiency of tea leaves compared to those of plants in natural ecosystems^24^. Although fertilization is an effective strategy to increase tea yield and improve tea quality, improper fertilization practices, such as excessive N or P application, often result in N-P imbalances, which hinders tea growth, reduce tea quality, and ultimately lead to economic losses^25^. Therefore, scientific fertilization and rational supplementation are crucial for improving nutrient use efficiency and enhancing tea quality. In tropical tea plantations like our study site (Hainan Island), heavy rainfall (> 1800 mm/year) and severe leaching necessitate organic amendments as the dominant fertilization strategy, contrasting with chemical-fertilizer-dependent systems in drier regions. While chemical fertilization effects are well-documented, organic fertilizer impacts on leaf stoichiometry under high-leaching conditions remain unquantified—a knowledge gap critical for sustainable management in tropical agricultural system.

To elucidate the poorly understood effects of increased organic fertilizer on the leaf C, N, and P contents and their stoichiometry in tropical tea plantation, we conducted an experiment on organic fertilizer application at the black tea plantation demonstration base in Shuiman township, Wuzhishan City, Hainan Island, China. The demonstration base mainly grows two varieties of black tea, namely *Camellia sinensis* var. *assamica* (CSA) and *Camellia sinensis* var. *sinensis* (CSS). Due to the large amount of rainfall and severe leaching in this area, the utilization rate of chemical fertilizers is low, and organic fertilizers have become one of the main fertilizers for local tea cultivation. To compensate for the soil nutrients lost due to leaching as much as possible, we carried out the organic fertilizer addition experiment to access leaf C, N, and P contents and C: N:P stoichiometry in mid-June. Our aims were to: (1) examine how increased organic fertilizer application affects leaf C, N, and P contents, and their stoichiometric ratios (C: N, C: P, and N: P) of CSA and CSS; and (2) identify the key soil physicochemical factors (e.g., soil organic C [SOC], pH, and soil moisture content [SMC]) that mediate these fertilization-induced changes in leaf C, N, and P contents and their stoichiometry.

## Materials and methods

### Study sites

**Fig. 1:**
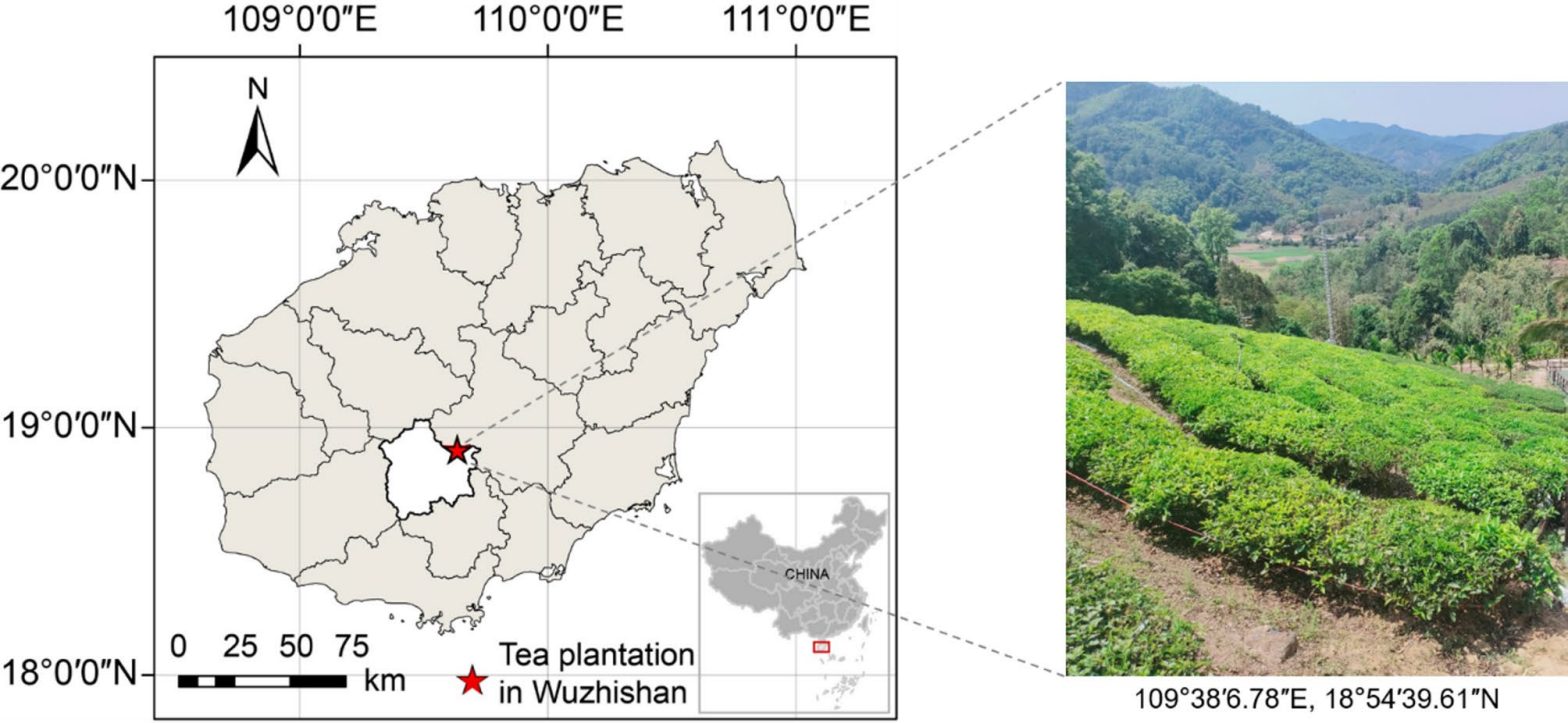
The geographical location of the sampling site.

This study was conducted at the black tea plantation demonstration base (18°54′39″N, 109°38′06″E) in Shuiman township, Wuzhishan City, Hainan Island, China (Fig. 1). The region is characterized by the tropical monsoon oceanic climate, with the mean annual temperature of 22.4 °C and annual precipitation ranging from 1800 to 2000 mm. Hainan Island has abundant rainfall, with a large variation range and distinct dry and wet seasons. The rainy season lasts from May to October, during which the rainfall accounts for approximately 80–90% of the annual rainfall. The dry season is from November to April of the following year, with the rainfall accounting for about 10–20% of the annual rainfall. The soil type is mainly red loam, characterized by a relatively high content of iron and aluminium oxides, a sticky texture, and good water retention capacity. Due to its specific soil type, as well as the climatic characteristics of high temperature and heavy rainfall, the coupling of soil nutrient elements with iron and aluminium cations in this area is intensified, resulting in intense leaching of the soil, rapid loss of nutrients, and severe nutrient limitations (especially P limitation).

The demonstration base has been operating normally for more than ten years. The routine management of the tea plantations primarily includes fertilization, weeding, and tea-picking. In November (dry season) of each year, approximately 250 g sheep manure organic fertilizer (produced by Hainan Organic Fertilizer Co., Ltd.) are applied using a hole application method at 0.5-meter intervals between each row of tea plants. The sheep manure organic fertilizer had the following characteristics (Table 1): moisture content 32.4%, pH 7.92, organic matter 40.91%, total nitrogen 0.793%, total phosphorus 1.16%, C: N ratio 23.7 (calculated from organic C and total N), water-soluble P 0.42%, NH_4_^+^-N 0.38% and NO_3_^−^-N 0.09% (determined by KCl extraction), and decomposition state- semi-decomposed (visible straw fragments < 2 cm). To replenish nutrients lost through leaching, a 20 × 20 m plot was established in tropical plantations of CSA and CSS black tea. The experiment also aimed to assess whether applying organic fertilizer during the rainy season would enhance the nutrient content of tea leaves. Sheep manure organic fertilizer was applied within the plot using the same fertilization method in mid-May 2023 (rainy season). The experimental plots (20 m × 20 m) were established on terraced fields with moderately sloping terraces (10–15°). To mitigate erosion and nutrient leaching, the terraces incorporated drainage ditches parallel to contour lines, and tea rows were planted perpendicular to the slope direction.

**Table 1:** Summary of soil physicochemical properties in the unfertilized and fertilized tea plantation of CSA and CSS.

Variables	CSA		CSS		Sheep manure fertilizer
	Unfertilized	Fertilized	Unfertilized	Fertilized	
SOC (g/kg)	21.74 ± 0.29b	23.42 ± 0.34a	21.04 ± 0.75b	25.09 ± 0.72a	–
Organic matter (g/kg)	–	–	–	–	324
Total N (g/kg)	1.36 ± 0.05b	1.96 ± 0.02a	1.99 ± 0.05b	2.18 ± 0.06a	7.93
Total P (g/kg)	0.49 ± 0.02b	0.79 ± 0.01a	0.56 ± 0.01b	0.62 ± 0.02a	11.60
C: N ratio	16.67 ± 0.68a	11.99 ± 0.18b	10.56 ± 0.23b	11.54 ± 0.20a	23.70
C: P ratio	47.66 ± 2.87a	29.77 ± 0.62b	38.34 ± 1.73a	41.06 ± 0.88a	16.20
N: P ratio	3.07 ± 0.22a	2.48 ± 0.03b	3.63 ± 0.14a	3.58 ± 0.09a	0.68
pH	5.92 ± 0.02a	4.45 ± 0.02b	5.57 ± 0.06a	4.58 ± 0.08b	7.92
SMC (%)	20.06 ± 1.10b	44.18 ± 1.02a	19.92 ± 0.48b	34.03 ± 0.33a	32.40
NO_3_^−^-N (mg/kg)	10.78 ± 0.64a	5.30 ± 0.36b	8.62 ± 0.81a	5.39 ± 0.51b	900
NH_4_^+^-N (mg/kg)	12.68 ± 0.90b	19.29 ± 1.06a	13.86 ± 1.50b	22.45 ± 1.62a	3800
SAP (mg/kg)	5.99 ± 0.43b	8.21 ± 0.63a	9.43 ± 0.74a	11.56 ± 1.01a	–
Water-soluble P (mg/kg)	–	–	–	–	4200

Note: Within the same line, different lowercase letters indicate a significant difference in the same edaphic variable between unfertilized and fertilized tea plantations of CSA and CSS (*P* < 0.05, t-test);

CSA, *Camellia sinensis* var. *assamica*; CSS, *Camellia sinensis* var. *sinensis*; SOC, soil organic carbon; SMC, soil moisture content; NO_3_^−^-N, nitrate nitrogen; NH_4_^+^-N, ammonium nitrogen; SAP, soil available phosphorus.

“–” indicates the absence of the data.

### Sampling and measurement

Before the application of organic fertilizer, samples of soil and leaves from both CSA and CSS plantations were collected. Ten non-rhizosphere soil samples at a depth of 0–20 cm were randomly collected (avoiding fertilization pits) and placed in self-sealing bags. The tea leaves at the corresponding positions were collected, packed in envelopes, and transported back to the laboratory as soon as possible together with soil samples. Six months after the application of sheep manure organic fertilizer during the rainy season, in November, soil samples and corresponding leaf samples were collected again at approximately the same locations as before, using the same sampling method. This sampling was conducted prior to the dry-season fertilization. We collected a total of 40 soil and 40 leaf samples. The soil samples brought back to the laboratory were divided into two parts. One part was stored at 4 °C for measuring ammonium nitrogen (NH_4_^+^-N), and nitrate nitrogen (NO_3_^−^-N), the other part was air-dried, ball-milled, sieved (< 2 mm) and homogenized for physicochemical analyses. After the leaves were deactivated in an oven at 105 °C (48 h), they were dried in an oven at 70 °C to a constant weight, ground, and used for the determination of C, N, and P contents.

Leaf C and N were measured by an elemental analyzer (Elementar Vario MAX, Germany), and P was determined by the molybdenum-antimony antimony colorimetric method^26^. SMC was determined from the loss of mass after drying soil samples at 105 °C for 48 h. Soil pH was measured in a 1:2.5 (w/v) aqueous solution using a pH meter (Brand, Germany). SOC was measured using dichromate oxidation method^27^. After H_2_SO_4_-HClO_4_ digestion, soil total N and total P were determined using the Kjeldahl and molybdate blue colorimetric method, respectively^26,28^. Soil nitrate nitrogen (NO_3_^−^-N) and ammonium nitrogen (NH_4_^+^-N) were extracted by 1 mol L^− 1^ KCl at 25 °C and determined on the continuous flow autoanalyzer 3^29^ (AA3). Soil available P (SAP) was estimated according to Bray-1 method^30^.

### Data analysis and statistics

We assessed the normality of the data using the Shapiro-Wilk test and evaluated the homogeneity of variances across different groups using Levene’s test. To detect the significant differences in leaf C, N, and P content and their stoichiometric ratios, and soil physicochemical properties between unfertilized and fertilized treatments in the CSA and CSS plantations, the t-test was conducted. Pearson correlation test was carried out to investigate the relationships between soil physicochemical properties and leaf C, N, and P contents, as well as their stoichiometric ratios in the unfertilized and fertilized CSA and CSS plantations. We used the partial least squares path model (PLS-PM) to evaluate the relationships among treatment (unfertilized and fertilized), soil physical and chemical properties, soil nutrient elements and their stoichiometric ratios, leaf C, N, and P contents, and their stoichiometric ratios in the CSA and CSS plantations. The bootstrap method (default iterations, 100) was used to validate the estimates of the path coefficients and coefficients of determination (R^2^). The overall predictive performance of the model was evaluated using the goodness-of-fit index.

All statistical analyses were performed in the R environment^31^ (v4.4.1), using *car* (v3.1.2), *ggplot2* (v3.5.1), *ggpubr* (v0.6.0), *ggpmisc* (v0.6.0), *rstatix* (v0.7.2), *corrplot* (v0.92), *psych* (v2.4.6.26), and *plspm* (v0.5.1) packages.

## Results

### Soil physicochemical properties in the unfertilized and fertilized CSA and CSS plantations

Fertilization significantly increased SOC, soil total N, and P contents in both CSA and CSS plantations, while significantly reducing soil C: N, C: P, and N: P ratios in the CSA plantation (Table 1). Although fertilization significantly increased soil C: N ratio of CSS plantation, it had no significant effect on soil C: P and N: P ratios (Table 1). Additionally, fertilization significantly decreased soil pH and NO_3_^−^-N of CSA and CSS plantations, but significantly increased NH_4_^+^-N (Table 1). We also found that the SMC in both fertilized CSA and CSS plantations was significantly higher than that in the unfertilized ones, which may be associated with the abundant rainfall during the rainy season. For SAP, fertilization significantly increased SAP content in the CSA plantation, but had no significant effects on it in the CSS plantation (Table 1).

### Leaf C, N, and P contents and their stoichiometry in the unfertilized and fertilized CSA and CSS

**Fig. 2:**
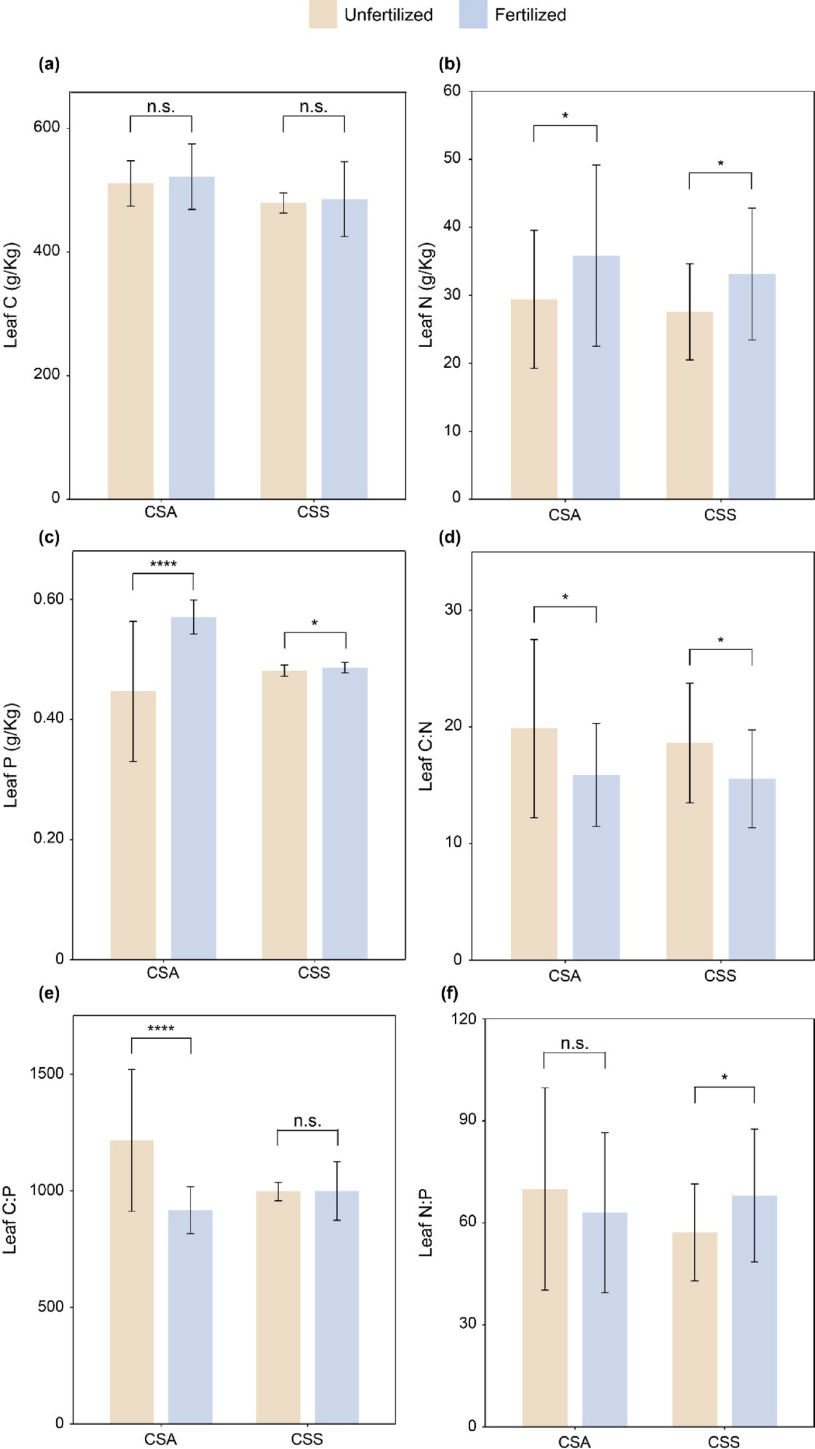
(**a**) Leaf C, (**b**) N, (**c**) P, (**d**) C: N, (**e**) C: P and (**f**) N: P of *Camellia sinensis* var. *assamica* and *Camellia sinensis* var. *sinensis*. CSA, *Camellia sinensis* var. *assamica*; CSS, *Camellia sinensis* var. *sinensis*. The asterisks above the mean ± SE indicate significant differences between unfertilized and fertilized CSA, as well as between unfertilized and fertilized CSS at *P* < 0.05 using t-test. Significance levels: *, *P* < 0.05; ***, *P* < 0.001; n.s., non-significant (*P* > 0.05).

Our results indicated that fertilization did not significantly increase leaf C content of CSA and CSS, but it significantly increased leaf N and P contents for both species (Fig. 2a and c). Fertilization significantly decreased leaf C: N ratio of CSA and CSS and leaf C: P ratio of CSA, but significantly increased leaf N: P ratio of CSS (Fig. 2d,f). Our results also showed that fertilization had no significant effect on leaf C: P ratio of CSS and leaf N: P ratio of CSA (Fig. 2e,f).

### The main influencing factors of leaf C, N, and P contents and their stoichiometry in the unfertilized and fertilized CSA and CSS

**Fig. 3:**
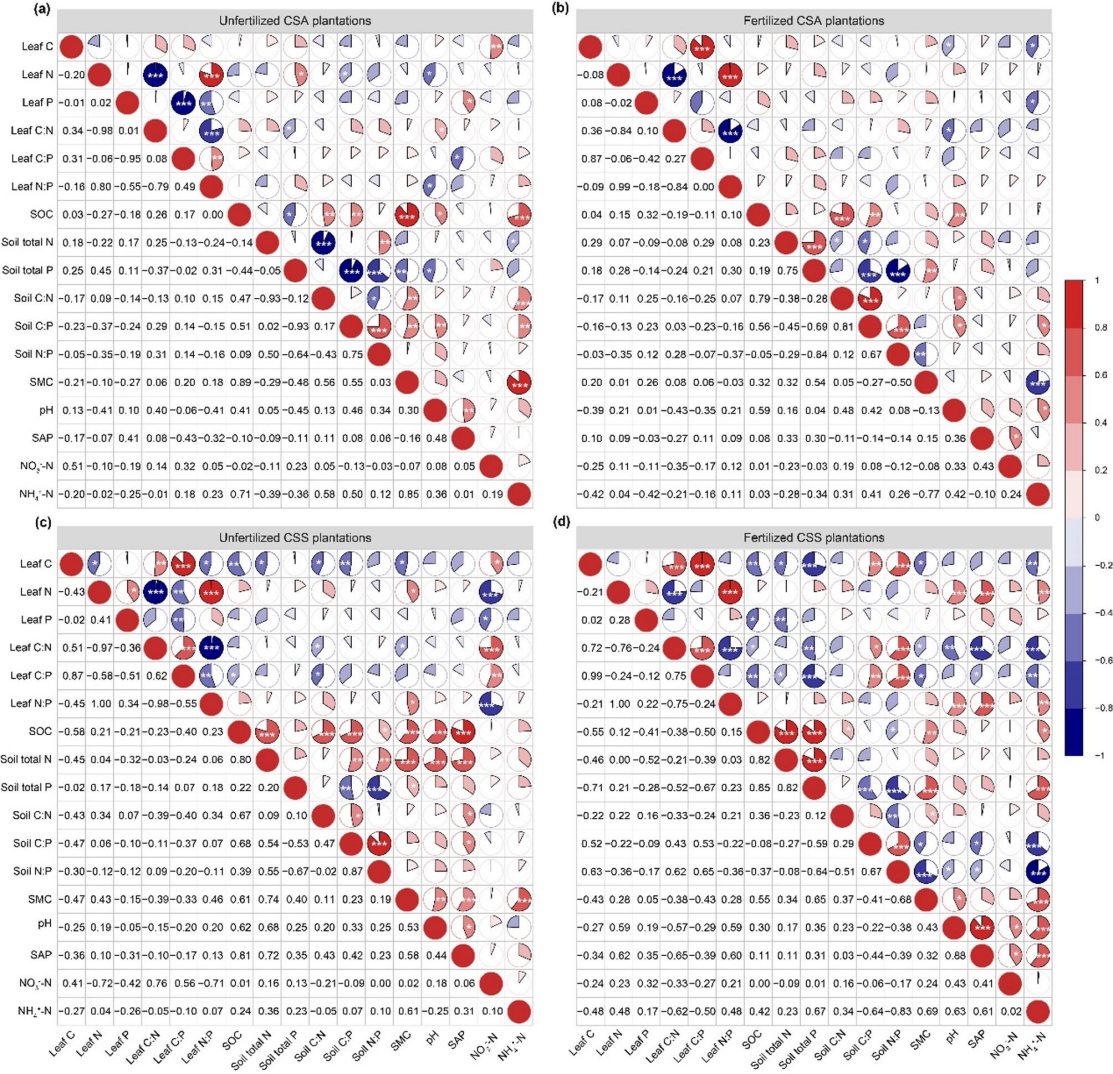
Pearson correlation coefficients among various variables of (**a**,**c**) unfertilized and (**b**,**d**) fertilized tea plantations of *Camellia sinensis* var. *assamica* and *Camellia sinensis* var. *sinensis*. The pie charts in the upper right indicate correlation strength, and the numbers in the lower left are the correlation coefficient. CSA, *Camellia sinensis* var. *assamica*; CSS, *Camellia sinensis* var. *sinensis*; SOC, soil organic carbon; SMC, soil moisture content; NO_3_^−^-N, nitrate nitrogen; NH_4_^+^-N, ammonium nitrogen; SAP, soil available phosphorus. Significance levels: ***P < 0.001, **P < 0.01; *P < 0.05.

In the unfertilized CSA tea plantation, soil NO_3_^−^-N was positively correlated with leaf C content (*r* = 0.51, *P* < 0.01; Fig. 3a). Soil pH showed the significant correlation with leaf N content (*r* = − 0.41, *P* < 0.05), C: N (*r* = 0.40, *P* < 0.05) and N: P ratios (*r* = − 0.41, *P* < 0.05; Fig. 3a). SAP was positively correlated with leaf P content (*r* = 0.41, *P* < 0.05) and negatively correlated with C: P ratio, respectively (*r* = − 0.43, *P* < 0.05; Fig. 3a). In the fertilized CSA tea plantation, soil NH_4_^+^-N was negatively correlated with leaf C (*r* = − 0.42, *P* < 0.05) and P content (*r* = − 0.42, *P* < 0.05; Fig. 3b). Soil pH showed the negative correlation with leaf C content (*r* = − 0.39, *P* < 0.05) and C: N ratio (*r* = − 0.43, *P* < 0.05; Fig. 3b). In the unfertilized CSS tea plantation, soil NO_3_^−^-N was positively correlated with leaf C content (*r* = 0.41, *P* < 0.05), C: N (*r* = 0.76, *P* < 0.001), and C: P (*r* = 0.56, *P* < 0.01), while showing negative correlations with leaf N (*r* = − 0.72, *P* < 0.001), P content (*r* = − 0.42, *P* < 0.05), and N: P (*r* = − 0.71, *P* < 0.001; Fig. 3c). In the fertilized CSS tea plantation, soil NH_4_^+^-N was negatively correlated with leaf C content (*r* = − 0.48, *P* < 0.01), C: N (*r* = − 0.62, *P* < 0.001), and C: P (*r* = − 0.50, *P* < 0.01), but positively correlated with leaf N content (*r* = 0.48, *P* < 0.01) and N: P (*r* = 0.48, *P* < 0.01; Fig. 3d).

**Fig. 4:**
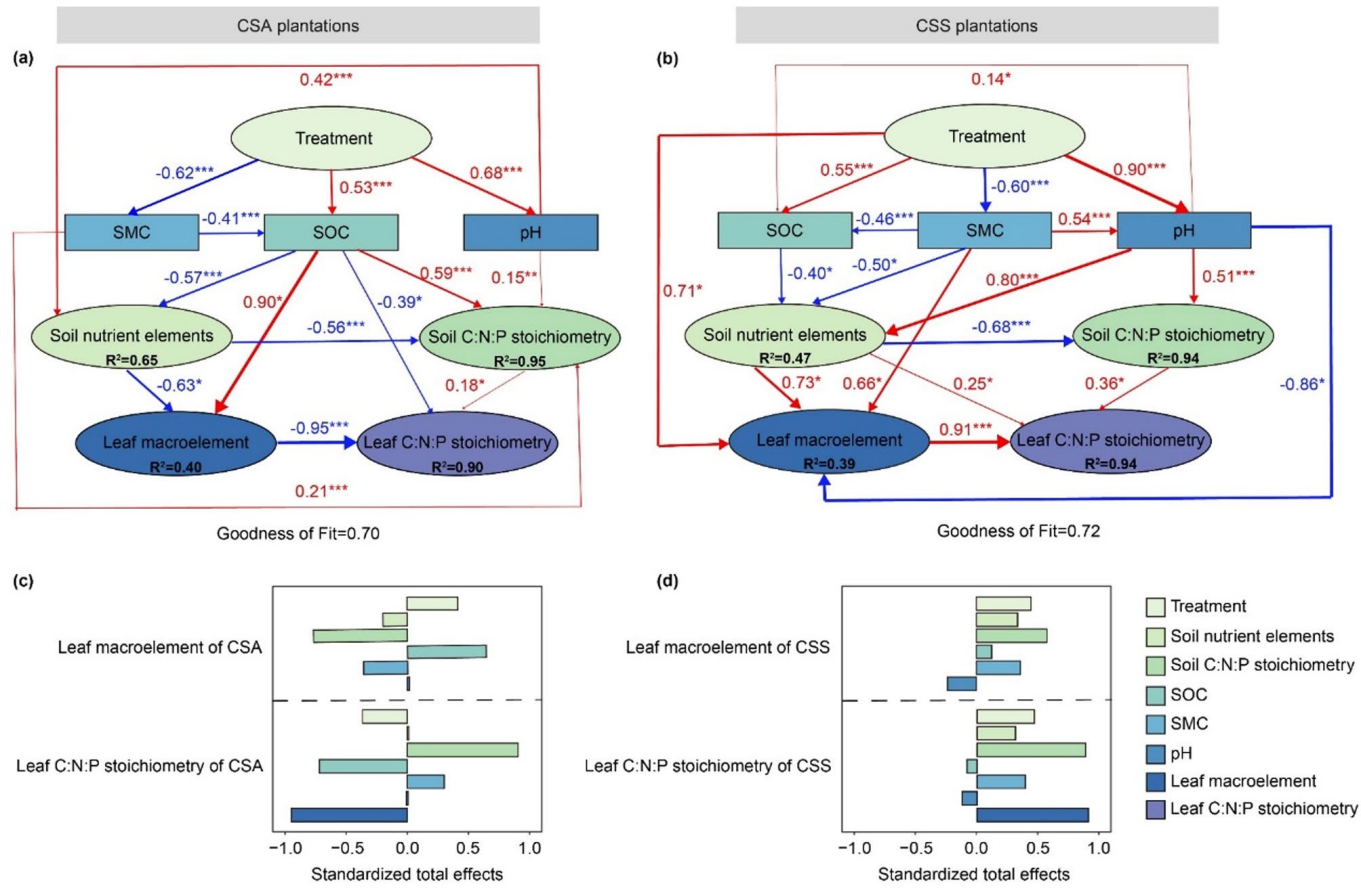
Partial least squares structural equation modeling (PLS-SEM) analysis of the relationships between treatment, soil properties, and leaf C, N, and P content and their stoichiometry of (**a**,**c**) *Camellia sinensis* var. *assamica* and (**b**,**d**) *Camellia sinensis* var. *sinensis*. Standardized path coefficients representing the effect sizes of potential causal variables are indicated by numbers adjacent to arrows. The width of arrows is proportional to the potential causal effect between variables. The red arrows indicate positive effects, and the blue arrows indicate negative effects. The numbers in the ellipse of response variables denote the explained variance (R^2^). Colors of rectangles and ellipses indicate different variable types. The variables in the rectangles represent observed variables, while the variables in the ellipses represent latent variables. The observed variables of ‘Treatment’ are unfertilized and fertilized. The observed variables of ‘Soil nutrient elements’ are the contents of soil total N, NO_3_^−^-N, NH_4_^+^-N, total P, and available P. The observed variables of ‘Soil C: N:P stoichiometry’ are soil C: N, C: P, and N: P. The observed variables of ‘Leaf macroelement’ are leaf C, N, and P contents. The observed variables of ‘Leaf C: N:P stoichiometry’ are leaf C: N, C: P, and N: P. CSA, *Camellia sinensis* var. *assamica*; CSS, *Camellia sinensis* var. *sinensis*. SOC, soil organic carbon; SMC, soil moisture content. The goodness-of-fit was used to assess the model. Significance levels: ***, *P* < 0.001; **, *P* < 0.01; *, *P* < 0.05.

The fertilization treatment did not directly affect the leaf C, N, and P contents and their stoichiometry of CSA, but rather influenced them indirectly by affecting soil physical and chemical properties (such as SMC, SOC, and soil pH; Fig. 4a). In contrast, the fertilization treatment directly influenced the leaf C, N, and P contents of CSS, thereby affecting their stoichiometry (Fig. 4b). The standardized total effect of fertilization treatments on the leaf C, N, and P contents of CSA was positive, whereas its total effect on their stoichiometry was negative (Fig. 4c). Unlike this, the fertilization treatment had a positive total effect on both the leaf C, N, and P contents and their stoichiometry of CSS (Fig. 4d). Soil C: N:P stoichiometry was identified as the primary factor influencing the leaf C, N, and P contents of CSA and CSS, while the leaf C, N, and P contents were the most direct and primary factors influencing their stoichiometry (Fig. 4c and d).

## Discussion

### Different responses of leaf C, N, and P contents and their stoichiometry of CSA and CSS to increased organic fertilizer

As our results demonstrated, the application of organic fertilizer during the rainy season significantly increased leaf N and P contents of both CSA and CSS, while C content showed no significant change (Fig. 2a and c). Although the increase in leaf P content of CSS under organic fertilizer treatment was statistically significant, the absolute difference in mean values was relatively small. This suggests that while the fertilizer may have triggered a measurable physiological response, the magnitude of change might be limited in terms of biological relevance. Therefore, caution should be taken when interpreting the ecological significance of this result. In addition, the exceptionally low leaf P content (0.45–0.60 g/kg) and elevated N: P ratios (57–70; Table S1) reveal profound P limitation in our study site. This aligns with Hainan’s highly weathered tropical soils, where iron/aluminum oxides immobilize P^32^. While organic fertilization increased leaf P by 25–33% (Fig. 2c), absolute P levels remained suboptimal compared to the typical range. This underscores P limitation as a critical factor constraining tropical tea productivity–a novel insight with significant management implications.

Notably, the application of organic fertilizer not only significantly increased soil total N and P contents in the CSA and CSS tea plantations but also enhanced their SOC (Table 1), which consistent with previous studies^33–35^. Sheep manure organic fertilizer is rich in organic matter and nutrients^36^. Applying sheep manure organic fertilizer can increase soil total C and nutrients (such as N, P, and potassium), rejuvenate and activate old organic matter, enhance soil fertility, and subsequently lead to increased leaf N and P contents^35^. However, sheep manure organic fertilizer significantly increased SOC in the CSA and CSS tea plantations, but it did not have a significant effect on their leaf C content. There are at least three potential reasons for the inconsistencies in the effects of sheep manure organic fertilizer on SOC and leaf C content. First, the sources of C for soil and plants are different. SOC primarily originates from the applied organic fertilizer and decomposition of soil organic matter, whereas leaf C is primarily fixed from atmospheric carbon dioxide through photosynthesis^37^. There is no direct connection between these two C pools. Second, nutrient limitation. Both CSA and CSS experienced strong P limitation before and after fertilization (leaf N: *P* > 16; Fig. 2f). Due to the severe P limitation, even with increased SOC, tea plants may not be able to fully utilize the additional C resources, resulting in no significant change in leaf C content. Third, C allocation and utilization. Under P limitation, plants tend to allocate the C fixed through photosynthesis preferentially to the roots^38^ and to symbiotic associations with arbuscular mycorrhizal fungi^39^ (AMF). Root expansion and AMF hyphal networks help plants more effectively acquire limited P from soil through direct exploration and enzymatic mineralization, respectively–an adaptive strategy to P limitation^40,41^. This C investment in AMF symbiosis represents a significant sink (up to 20% of photosynthates), which may further constrain carbon allocation to leaf tissues. Therefore, the increase in SOC may not directly impact leaf C content of CSA and CSS.

Comparatively, the stoichiometric responses of leaf C, N, and P of CSA to increased organic fertilizer application were consistent with those of soil C, N, and P in the CSA tea plantation. Specifically, sheep manure application reduced both leaf and soil C: N, C: P, and N: P ratios in CSA, but this pattern was not observed in CSS (Fig. 2d and f; Table 1). This may be attributed to these differences in nutrient utilization strategies and physiological mechanisms between these two types of tea plants. Compared to CSA, CSS has smaller leaves, and the reduced leaf area minimizes water and nutrient loss through transpiration^42^. Additionally, the relatively slower growth rate of CSS implies a lower nutrient demand and higher nutrient use efficiency, making it easier to sustain stable growth under limited nutrient supply^43^.

Despite its C: N ratio of 23.7, the sheep manure contained high labile N and P fractions (Table 1). Under tropical conditions (high temperature and humidity), these properties collectively enhanced microbial activity and accelerated mineralization of organic N and P, and increasing the availability of these nutrients^44^. As a result, tea plants absorb more N and P, leading to higher leaf N and P relative to C, significantly reducing leaf C: N and C: P ratios (Fig. 2d and e). Unlike C and N, the primary source of P is the weathering of the parent material through physical processes^45^. External input of P (e.g., fertilization) can significantly enhance soil and leaf P content^46^. As P is the limiting element for plants in the study region, its impact far outweighs that of N. Consequently, the application of organic fertilizer (e.g., sheep manure) reduced leaf N: P ratio of CSA (Fig. 2f). However, the leaf N: P ratio of CSS increased with organic fertilizer application (Fig. 2f), which may be attributed to the unique physiological characteristics and ecological adaptability of CSS.

### Fertilization treatments affect leaf C, N, and P contents of CSA and CSS through distinct pathways

In the unfertilized tea plantation, leaf C, N, and P contents and their stoichiometry of CSA and CSS were significantly correlated with soil NO_3_^−^-N content (Fig. 3a and c). In contrast, in the fertilized tea plantation, leaf C, N, and P contents and their stoichiometry of CSA and CSS were significantly correlated with soil NH_4_^+^-N content (Fig. 3b and d). This difference may be attributed to the high C: N ratio of the applied organic fertilizer. The moderately high C: N ratio (23.7) of the applied fertilizer suggests gradual mineralization, explaining the sustained NH_4_^+^-N release observed in fertilized soils (Table 1). This aligns with the significant correlation between soil NH_4_^+^-N and leaf stoichiometry under fertilization (Fig. 3b and d). Specifically, the application of sheep manure organic fertilizer decreased soil NO_3_^−^-N content while increased NH_4_^+^-N content. Consequently, in the unfertilized soils, NO_3_^−^-N might be the primary available N source, which explains its significant correlation with leaf C, N, P contents and their stoichiometry. In the fertilized soils, due to the decomposition of sheep manure organic fertilizer and N transformation processes, NH_4_^+^-N became the dominant available N source. Additionally, the decrease in soil pH following organic fertilizer application (Table 1) likely resulted from enhanced nitrification of NH_4_^+^ derived from mineralization. Nitrification (NH_4_^+^ → NO_3_^−^) produces H^+^ ions, directly acidifying the soil solution^47^. The low pH subsequently inhibited ammonia-oxidizing archaea and bacteria^48,49^, further suppressing nitrification rates and favoring NH_4_^+^ accumulation. This shift in N transformation pathways influenced plant N uptake and leaf stoichiometric regulation.

The total effects of fertilization treatments on the leaf C, N, and P contents of CSA and CSS was positive (Fig. 4c and d), which were consistent with the earlier results (Fig. 2a and c). The total effect of fertilization on the leaf C: N:P stoichiometry of CSA was negative (Fig. 4c), while for CSS, the total effect is positive (Fig. 4d), which also aligned with the previous findings (Fig. 2d and e). These results further suggests that in the response of leaf C: N:P stoichiometry of CSS to fertilization, the influence of N: P ratio on the latent variable of C: N:P stoichiometry is greater than that of C: N. This is because, compared to C, N and P are functional elements for plants, whose availability is constrained by soil nutrient supply and are more likely to become limiting nutrients^50,51^. According to the law of the minimum, even when other nutrients are adequately supplied, plant growth is constrained by the nutrient in shortest supply, which in this case is P^52^.

### Limitations and uncertainties

Although this study provides important insights into the use strategies of soil fertilizers in tropical tea plantations, we have to acknowledge two limitations. First, the limited data on soil physicochemical properties, such as soil texture, structure, and cation exchange capacity, may hinder the exploration of the driving factors of C, N and P contents and their stoichiometry in tropical tea plantation. Second, it is undeniable that the duration of our increased organic fertilizer experiment was somewhat short, which may have resulted in the effects of increased organic fertilizer on the content of soil nutrient elements, stoichiometry, and homeostasis of tea plant being less apparent. Therefore, more data on explanatory variables are clearly required, and longer-term increased organic fertilizer are essential for further understanding the mechanisms underlying the response of tropical tea plantation soils and tea plants to organic fertilizer application. In addition, while topography may influence nutrient distribution, the terraced design of our study site minimized spatial heterogeneity. Future studies should incorporate digital elevation models to quantify micro-topographic effects on fertilizer dynamics.

### Implications and future directions

These findings have important implications for nutrient management in tropical tea plantations. The greater responsiveness of CSS to organic fertilization, particularly in terms of increased leaf N and P, suggests that fertilization strategies may need to be tailored to varietal traits and targeted supplementary application of P fertilizers. While our study does not define the optimal application rate, the results indicate that moderate increases in organic fertilizer can improve nutrient status without excessive nutrient accumulation in leaves, particularly in CSS. This highlights the potential for fine-tuning fertilization regimes to enhance nutrient use efficiency and reduce environmental risks associated with over-fertilization. Future research should explore long-term effects of different application rates, interactions with microbial processes, and the cost-effectiveness of variety-specific management strategies under field conditions.

## Conclusions

Our results suggest that sheep manure organic fertilizer significantly increased the leaf N and P contents of CSA and CSS in the tropical tea plantation, while it had no significant effect on the leaf C content. Notably, the organic fertilizer application significantly increased the contents of SOC, soil total N, and total P, while decreasing the soil C: N, C: P, and N: P ratios in the CSA, but not in the CSS. The inconsistencies in the responses of soil and plant C content to the application of sheep manure organic fertilizer may be attributed to differences in C sources between soil and plants, as well as the unique C allocation and utilization strategies of tea plants under P-limited conditions. This study found that in the unfertilized tea plantation, the leaf C, N, and P contents and their stoichiometry of CSA and CSS were significantly correlated with soil NO_3_^−^-N content, whereas in the fertilized tea plantation, they were significantly correlated with soil NH_4_^+^-N content. Furthermore, the present study suggested that the differences in the pathways through which sheep manure organic fertilizer influences the leaf C, N, P contents of CSA and CSS may be ascribed to the distinct leaf physiological characteristics of these two tea plant species.

Given the ecological and agricultural importance of tea plantations in tropical regions, understanding how organic fertilizer influences leaf C, N, P contents and their stoichiometric ratios provides critical insights into optimizing fertilization practices and improving nutrient management strategies. This study highlights conventional organic fertilization cannot fully alleviate P deficits in highly weathered soils, necessitating targeted P supplementation strategies. Future research should explore the long-term effects of organic amendments and their interactions with soil microbial processes to better inform adaptive nutrient management in diverse tea-growing regions.

## Electronic supplementary material

Below is the link to the electronic supplementary material.

Supplementary Material 1

## Data Availability

The datasets used and/or analysed during the current study available from the corresponding author on reasonable request.
