# Supplementary material for: Increased organic fertilizer significantly increases leaf nitrogen and phosphorus but not carbon content in a tropical tea plantation
Source: Sci Rep. 2025 Jul 19;15:26249. doi: 10.1038/s41598-025-11057-z (PMC12276241; doi:10.1038/s41598-025-11057-z)
Supplement: Supplementary file 1 — Supplementary Material 1 [file 41598_2025_11057_MOESM1_ESM.docx]

**Increased organic fertilizer significantly increases leaf nitrogen and phosphorus but not carbon content in a tropical tea plantation**

*Jing-li Lu^1^*, *Ying Wang^1^*, *Di Li^1^*, *Qiu Yang^1^*, *Yamin Jiang^1^*, *Peng Wang^1^*, *Tianyan Su^1^*, *Geming Li^1^*, *Qian Shi^1^*, *Huai Yang^2,*^*, *Wenjie Liu^1,3,^*^*^, *Mengyang Fang^3^*

*^1^ Center for Eco-Environment Restoration Engineering of Hainan Province, School of Ecology, Hainan University, Haikou, 570228,* *People's Republic of China*

*^2^ Institute of Tropical Bamboo, Rattan & Flower, Sanya Research Base, International Center for Bamboo and Rattan, Sanya 572000, People's Republic of China*

*^3^ Sanya Tropical Ecosystem Carbon Source and Sink Field Scientific Observation and Research Station, Sanya 572022, People's Republic of China*

*Correspondence: Huai Yang

Email: yanghuai2008@163.com

Wenjie Liu

Email: [liuwj@hainanu.edu.cn](mailto:liuwj@hainanu.edu.cn)

Tel & Fax: + 08 98 66269468

**Table S1. Typical ranges of leaf nutrient contents in *Camellia sinensis*.**

| Variables | This study (Unfertilized) | This study (Fertilized) | Typical range in tea leaves | Citations |
| --- | --- | --- | --- | --- |
| Leaf C (g/kg) | CSA: 510.91±6.61  CSS: 479.44±3.08 | CSA: 521.98±9.99  CSS: 485.65±10.88 | 430–480 | Ruan et al., 2007 |
|  |  |  |  |  |
| Leaf N (g/kg) | CSA: 29.42±1.82  CSS: 27.57±1.33 | CSA: 35.84±2.52  CSS: 33.13±1.74 | 28–42 | Venkatesan et al., 2004 |
|  |  |  |  |  |
| Leaf P (g/kg) | CSA: 0.45±0.02  CSS: 0.48±0.002 | CSA: 0.60±0.005  CSS: 0.52±0.002 | 1.5–2.5 | Yin et al., 2021 |
|  |  |  |  |  |
| Leaf N:P | CSA: 69.98±5.34  CSS: 57.21±2.69 | CSA: 62.00±4.45  CSS: 68.07±3.51 | 14–22 (P-limited systems) | Güsewell, 2004 |

Note: CSA, *Camellia sinensis* var. *assamica*; CSS, *Camellia sinensis* var. *sinensis*.

**References**

Güsewell, S. N:P ratios in terrestrial plants: variation and functional significance. *New Phytol.* **164**, 243–266 (2004).

Ruan, J., Gerendás, J., Härdter, R. & Sattelmacher, B. Effect of nitrogen form and root-zone pH on growth and nitrogen uptake of tea (*Camellia sinensis*) plants. *Ann. Bot.* **99**, 301–310 (2007).

Venkatesan, S., Murugesan, S., Ganapathy, M. N. & Verma, D. P. Long‐term impact of nitrogen and potassium fertilizers on yield, soil nutrients and biochemical parameters of tea. *J. Sci. Food Agric.* **84**, 1939–1944 (2004).

Yin, X. L. et al. Effects of different management methods on carbon, nitrogen, and phosphorus contents and their stoichiometric ratios in tea plants. *Chin. J. Plant Ecol.* **45**, 749 (2021).
